# Supplementary material for: Failure Processes in Embedded Monolayer Graphene under Axial Compression
Source: Sci Rep. 2014 Jun 12;4:5271. doi: 10.1038/srep05271 (PMC4053737; doi:10.1038/srep05271)
Supplement: Supplementary Information — Supporting Information [file srep05271-s1.pdf]

Supporting Information

Failure Processes in Embedded Monolayer Graphene under Axial Compression

*Charalampos Androulidakis, Emmanuel N. Koukaras, Otakar Frank, Georgia Tsoukleri, Dimitris Sfyris, John Parthenios, Nicola Pugno, Konstantinos Papagelis, Kostya S. Novoselov, and Costas Galiotis**

1. Mathematical Analysis

According to the Euler stability criterion, the critical state for compression failure is reached when the work of the external forces equals the change in strain energy of the body[1,2]:

$\Delta T = \Delta U, \Delta U = \Delta U_b + \Delta U_f$. (S1)

Furthermore, the work of the compressive forces acting on the middle plane of the plate is given by[2]:

$$\Delta T = -\frac{1}{2}\int_A N_x \left(\frac{\partial u}{\partial x}\right)^2 dA, \qquad \text{(S2)}$$

where u is the out-of-plane displacement, A is the area of the plate (graphene) and N_x is the compressive force along the x-direction. The bending energy is given by[2]:

$$\Delta U_b = \frac{D}{2}\int_A \left\{ \left(\frac{\partial^2 u}{\partial x^2} + \frac{\partial^2 u}{\partial y^2} \right)^2 - 2(1-v)\left[\frac{\partial^2 u}{\partial x^2}\frac{\partial^2 u}{\partial y^2} - \left(\frac{\partial^2 u}{\partial x \partial y} \right) \right]^2 \right\} dA, \qquad \text{(S3)}$$

where D is the bending stiffness and v the Poisson ratio of graphene. The surrounding polymer matrix is assumed to contribute to the system through the deformation energy:

$$\Delta U_f = \frac{K_w}{2}\int_A u^2 dA, \qquad \text{(S4)}$$

where K_w is the Winkler's modulus. From the physical point of view the last term describes the interaction between the plate and the elastic foundation which is loaded as the plate bends at the critical point.

By equating the work of the external forces with the change in the strain energy we obtain:

$$\begin{aligned}
&\Delta T = \Delta U_b + \Delta U_W \rightarrow \\
&-\frac{1}{2}\int_A N_x \left(\frac{\partial u}{\partial x}\right)^2 dA = \qquad \text{(S5)} \\
&= \frac{D}{2}\int_A \left\{ \left(\frac{\partial^2 u}{\partial x^2} + \frac{\partial^2 u}{\partial y^2} \right)^2 - 2(1-v)\left[\frac{\partial^2 u}{\partial x^2}\frac{\partial^2 u}{\partial y^2} - \left(\frac{\partial^2 u}{\partial x \partial y} \right) \right]^2 \right\} dA + \frac{K_w}{2}\int_A u^2 dA.
\end{aligned}$$

For the out-of-plane displacement it is common to assume that it has a sinusoidal form[2-6] which models adequately the form of buckling that appears at the critical strain. For our purposes we make the following assumption for u:

$$u(x,y) = \sum_{m=1}^{\infty}\sum_{n=1}^{\infty} a_{mn} \sin\left(\frac{m\pi x}{l}\right)\sin\left(\frac{n\pi y}{w}\right), \qquad \text{(S6)}$$

where l, w is the length and the width of the flake, respectively. Under the above assumption we obtain:

$$\Delta U_b = \frac{\pi^4 lw}{8} D \sum_{m=1}^{\infty}\sum_{n=1}^{\infty} a_{mn}^2 \left(\frac{m^2}{l^2} + \frac{n^2}{w^2} \right)^2 , \qquad \text{(S7)}$$

$$\Delta T = \frac{\pi^2 w}{8l} N_{xx} \sum_{m=1}^{\infty}\sum_{=1}^{\infty} m^2 a_{mn}^2 , \qquad \text{(S8)}$$

$$\Delta U_f = \frac{K_w lw}{8} \sum_{m=1}^{\infty}\sum_{n=1}^{\infty} a_{mn}^2 . \qquad \text{(S9)}$$

Using the expression on eq 5 we obtain the critical force N_x:

$$N_x = \frac{\frac{\pi^4 lw}{8} D \sum_{m=1}^{\infty}\sum_{n=1}^{\infty} a_{mn}^2 \left(\frac{m^2}{l^2} + \frac{n^2}{w^2} \right)^2 + \frac{K_w lw}{8} \sum_{m=1}^{\infty}\sum_{n=1}^{\infty} a_{mn}^2}{\frac{\pi^2 w}{8l} N_{xx} \sum_{m=1}^{\infty}\sum_{=1}^{\infty} m^2 a_{mn}^2} . \qquad \text{(S10)}$$

Following the reasoning similar to reference 2, the critical force N_x, being a sum of positive quantities, is minimized when only one term α_{mn} is different than zero. In such a case we have:

$$N_x = \frac{\pi^2 l^2 D}{m^2} \left(\frac{m^2}{l^2} + \frac{n^2}{w^2} \right)^2 + \frac{l^2 K_w}{\pi^2 m^2} . \qquad \text{(S11)}$$

If we further make the physically plausible assumption[2] that there are several half waves in the direction of compression but only one half wave in the perpendicular direction (n=1) and use the formula $N_x=\varepsilon C$, where C is the tension rigidity, we finally arrive at the following expression for the critical strain for buckling:

$$\varepsilon_{cr} = \pi^2 \frac{D}{C} \frac{k}{w^2} + \frac{l^2}{\pi^2 C}\left(\frac{k_w}{m^2}\right), \qquad \text{(S12)}$$

where

$$k = \left(\frac{mw}{l} + \frac{l}{mw}\right)^2. \qquad \text{(S13)}$$

The determination of the half waves, *m*, stem from equating the force expression 11, for *m* and *m+1*:

$$N_m = N_{m+1} \qquad \text{(S14)}$$

This way we obtain the following equation for *m*:

$$m^2(m+1)^2 = \frac{l^4}{w^4} + \frac{l^4 K_w}{\pi^4 D} \qquad \text{(S15)}$$

The evaluation of the half wavelength and the amplitude relies on the inextensibility assumption. We assume that for small values of applied strain, the length of the specimen remains the same after buckling initiates. The wavelength that corresponds to the half-wave number is evaluated according to the formula:

$$\lambda = \frac{l\left(1-\varepsilon_{cr}\right)}{m} \qquad \text{(S16)}$$

The projection of the buckled length divided by the number of half waves, *m*, corresponds to the wave length. The length of the projection can be calculated by: $(l-\Delta l)$, figure S1 where $\Delta l = \varepsilon_{cr} l$.

The incompressibility constraint is a plausible assumption since we are at the regime of low strains (approximately ~-0.5 %). The line integral:

$$s = \int_{x_1}^{x_2} \left(1 + \left(\frac{\mathrm{d}u}{\mathrm{d}x}\right)^2\right)^{0.5} \mathrm{d}x = \int_0^{l(1-\varepsilon_{cr})} \sqrt{1 + \left[\frac{Am\pi}{l(1-\varepsilon_{cr})}\cos\left(\frac{m\pi\,\mathrm{x}}{l(1-\varepsilon_{cr})}\right)\right]^2} \mathrm{d}x \qquad \text{(S17)}$$

corresponds to the length of the flake. In the above relation u is the out-of-plane displacement that now takes the form:

$$u(x,y) = \sum_{m=1}^{m_{\max}} A\sin\left(\frac{m\pi x}{l}\right)\sin\left(\frac{\pi y}{w}\right), \quad \text{(S18)}$$

A its amplitude, while the term $(1\text{-}\varepsilon_{cr})$ describes the contractions due to buckling as the figures shows.

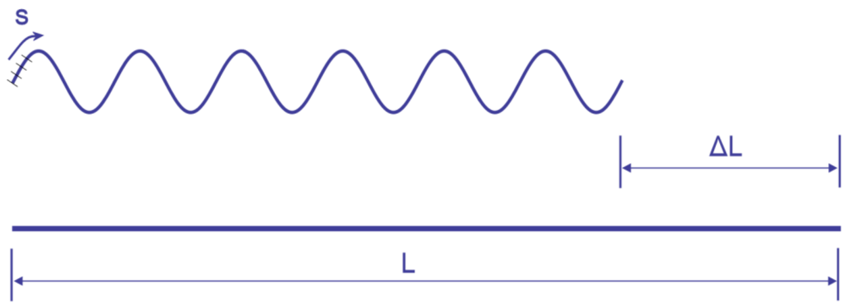

Figure S1. The initial length of the specimen and the length after buckling occurs.

2. Stress transfer from the PMMA to the graphene flake

We apply a compressive loading to the system graphene–PMMA. This kind of loading results to a shear stress at the interface between the graphene flake and the surrounding medium which is responsible for transferring the stress to the inclusion (graphene)[8]. The shear stresses require a specific length to reach the maximum value of the stress that is possible to be transferred from the polymer to the graphene. This is the transfer length L_t. So, if the length of the graphene is smaller than the critical transfer length, L_c ($L_c=2L_t$) then graphene is not stressed to the maximum value which is the externally applied stress through the flexure of PMMA beams. Indeed, only a fraction of the load is transferred to graphene.

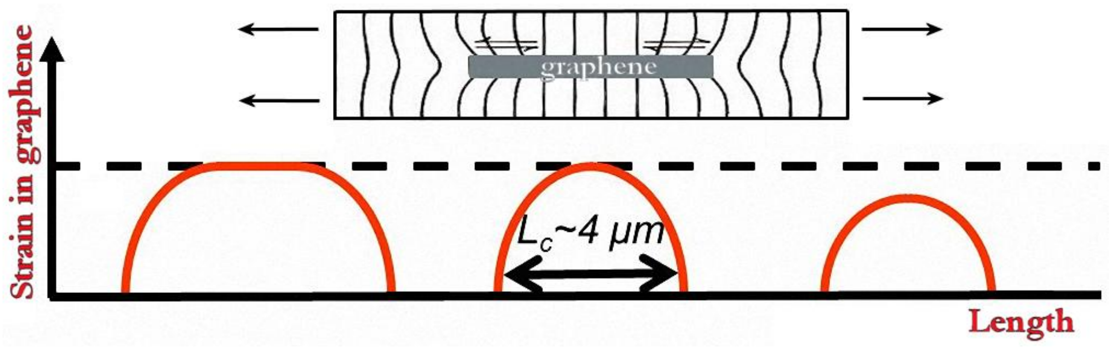

Figure S2. Building of stress transfer in graphene.

In the case of flakes with lengths not large enough compared to the required critical length (L_c), the strain/ stress that is transferred to the flake will never reach the maximum value (applied strain). Thus, the actual strain developed in the graphene must be corrected as it is not the same with the external strain. Our experimental results indicate that the flakes with length of up to about ~4 μm are not able to obtain the maximum values of the stresses applied to the system. This is observed by the slope of the curve Pos2D vs Strain (here the strain is that applied to the beam). In all these cases the slope is much smaller than the critical slope, ~60 cm^{-1}/% (estimate based on the present work as well as values from the literature, see Refs [9–11]). Thus, a simple correction can be implemented by shifting the Pos2D vs Strain slope near the origin (zero strain) and recalibrating the actual strain through the formula:

$$\varepsilon_{graphene} = \varepsilon_{applied}\left(\frac{\left(\frac{\partial \Delta \nu}{\partial \varepsilon}\right)_{measured}}{\left(\frac{\partial \Delta \nu}{\partial \varepsilon}\right)_{\max imum}}\right)_{T,\ \varepsilon=0} \quad \text{or } \varepsilon_{graphene}(\%) = \frac{\varepsilon_{applied}(\%)}{|60|}\left(\frac{\partial \Delta \nu}{\partial \varepsilon}\right)_{measured,\ \varepsilon=0} \qquad \text{(S20)}$$

3. Fitting

In figure 2 the fourth order polynomial *Pos2D*=2596 – 56.41ε– 29.54ε^2 + 13.16ε^3 + 4.01ε^4 is fitted to the data, is in closed form and captures the observed trend of the experimental data within the *full range* of strain levels under study. This was the lowest polynomial order that

yielded acceptable fitting of the data points at *both* the region near the origin and the region near failure point. An alternative approach would be to use two separate 2nd degree polynomials, one for each region of interest. Such polynomials, for example, would have the form $y=2595.8 – 56.1x – 33.2x^2$ and $y=2595.4 – 61.2x – 42.8x^2$ for the regions near the origin and at the failure point, respectively. These functions reproduce almost exactly our values for the slope at the origin and critical strain of failure.

4. Graphene–PMMA Interaction Potentials

Optimized structures

The optimized structures are shown in figure 7 of the manuscript. The structures have a helical pitch of 19.4 Å and 6.75 Å, an outer diameter of 10.1 Å and 23.2 Å, and a helical tilt angle (with respect to the axis of the main chain) of 28.6° and 78.7°, for the *i*-PMMA and *s*-PMMA chains respectively. The inner diameter of the *s*-PMMA helix is 13.8 Å which is substantially larger than the outer diameter of the *i*-PMMA helix and thus permits the formation of the 2:1 (*s*:*i*) PMMA stereocomplex. These values are in good agreement with the high-resolution atomic force microscopy measurements on the 2:1 (*s*:*i*) PMMA stereocomplex of Kumaki et al. who reported an outer *s*-PMMA helix tilt angle of 74°, a chain–chain lateral spacing of 24 Å and a helical pitch of 9.2 Å. Any difference from our theoretical values can be attributed to the influence of the internal *i*-PMMA double helix of the experimental stereocomplex structure.

The orientations of the monomers in the optimized polymer chains have been employed as a guide in selecting the most probable MMA monomer–coronene relative configurations for the PES scan, which are shown in figure S3. We denote these relative configurations of a MMA monomer and coronene as *h*-PC, in which the O–C (single) bond of a MMA monomer is horizontal, i.e. parallel to the coronene plane, and *v*-PC, for the case which the O–C (single)

bond of the MMA monomer is vertical, i.e. perpendicular to the coronene plane. The third configuration corresponds to an approximate reversed *h*-PC. A fourth configuration, not shown, corresponds to an *h*-PC with the monomer's backbone tilted by 30° with respect to the normal axis of the coronene plane.

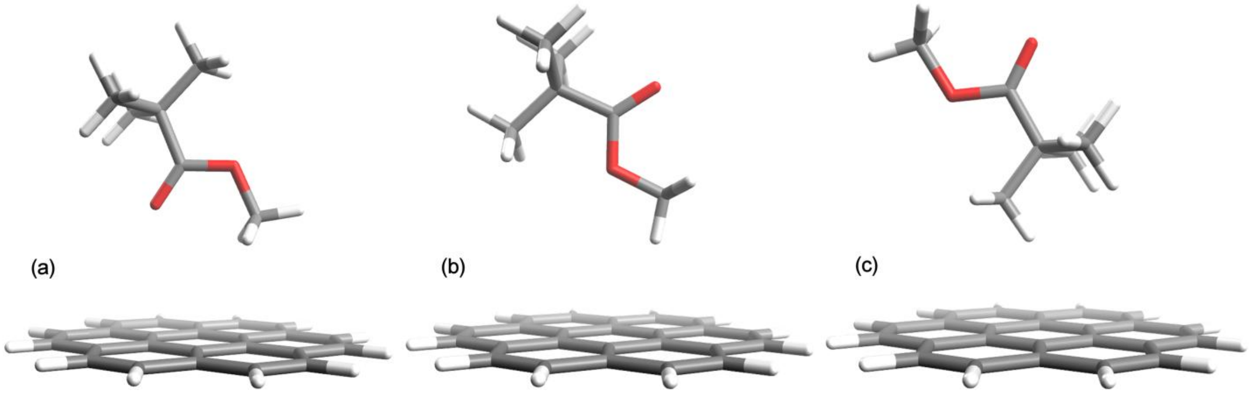

Figure S3. PMMA–coronene relative configurations used for the PES scans. The configurations correspond to the potentials (a) u_1, (b) u_2, and (c) u_3. A fourth configuration used is the same as (a) tilted by 30 degrees.

Primitive potentials

Compared to both the results from using the B2PLYPD functional as well as the SCS(MI)-MP2 method, the B97-D functional slightly overestimates the interaction energy (see figure S4a). A scaling factor of 1.11 is applied to the values obtained from the B97-D functional in order to match the results of the more accurate (but impractical for computational reasons) B2PLYPD functional and SCS(MI)-MP2 method. The primitive potentials are fitted to the scaled B97-D datasets. The values for the fitting parameters, σ, ε and C, for the primitive potentials, $u_{1–4}$, are, ε_1 = 18.98 kJmol^{-1}, σ_1 = 2.80778 Å, C_1 = 0.74371, ε_2 = 16.68 kJmol^{-1}, σ_2 = 2.99278 Å, C_2 = 0.68142, ε_3 = 21.59 kJmol^{-1}, σ_3 = 2.7788 Å, C_3 = 0.68782, and ε_4 = 19.09 kJmol^{-1}, σ_4 = 2.91439 Å, C_4 = 0.78586. The primitive potentials, $u_{1\text{-}4}(z)$, thus take the form:

$$u_1(z) = 4\times 18.98\left(\left(\frac{2.81}{z}\right)^{12\times 0.74} - \left(\frac{2.81}{z}\right)^{6\times 0.74}\right) \quad : PES1$$

$$u_2(z) = 4\times 16.68\left(\left(\frac{2.99}{z}\right)^{12\times 0.68} - \left(\frac{2.99}{z}\right)^{6\times 0.68}\right) \quad : PES2$$

$$u_3(z) = 4\times 21.59\left(\left(\frac{2.78}{z}\right)^{12\times 0.69} - \left(\frac{2.78}{z}\right)^{6\times 0.69}\right) \quad : PES3$$

$$u_4(z) = 4\times 19.09\left(\left(\frac{2.91}{z}\right)^{12\times 0.79} - \left(\frac{2.91}{z}\right)^{6\times 0.79}\right) \quad : PES4$$

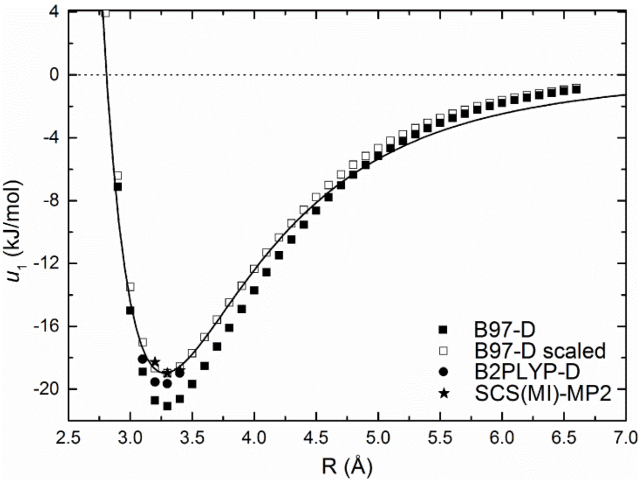

(a)

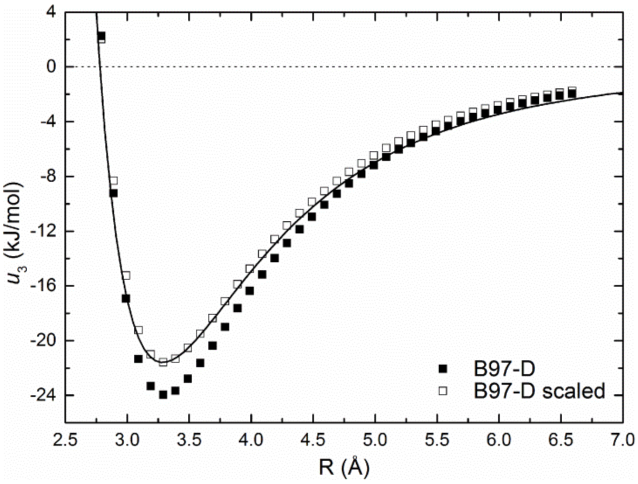

(b)

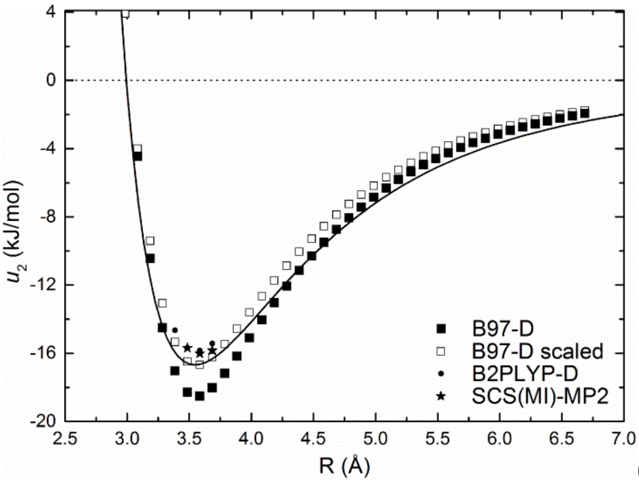

(c)

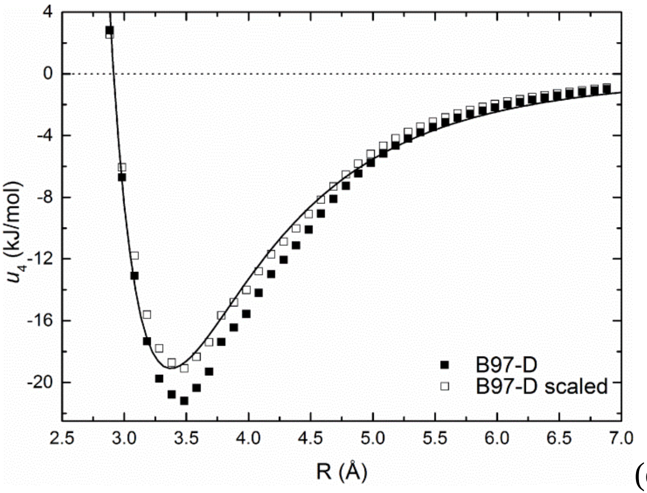

(d)

Figure S4. Potential energy surface scans for the configurations PMMA–Coronene shown in figure S3. *R* is the transverse distance of the PMMA carbon atom nearest to the coronene plane.

Two-parameter PES scan

We have additionally performed a two-parameter PES scan on a coronene with two MMA molecules, one on each of the coronene surfaces. The configuration corresponds to that used in PES1, and the scanning parameters are the distances R_1, R_2 between each monomer and the coronene plane. The resulting scan is shown in figure S5. The presence of the second MMA

molecule reduces the interaction very slightly, and the overall interaction energy is 1.2 % less compared to twice the interaction energy of the corresponding single monomer case.

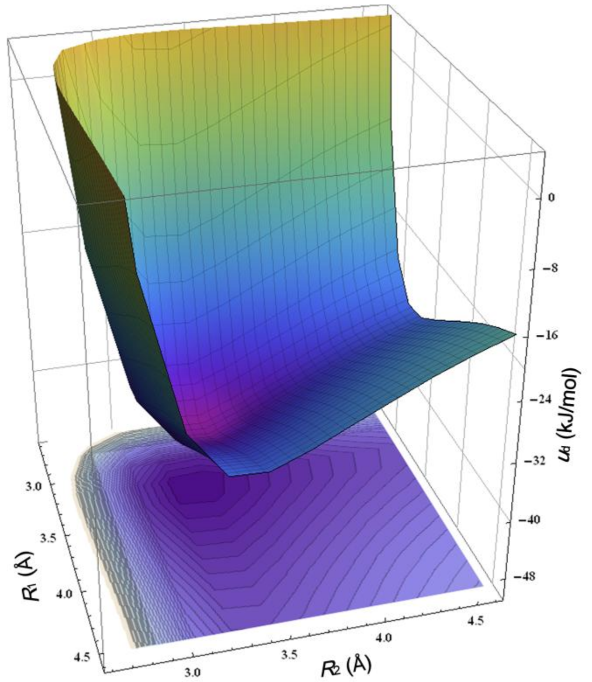

Figure S5. Two parameter potential energy surface scan corresponding to PMMA-coronene configurations as in PES1 with two MMA monomers, one on each side of coronene. The distances of each monomer from the coronene plane are denoted as R_1 and R_2.

Composite potentials

The final potentials have been constructed as a linear combination of the primitive potentials from the PES scans. The coefficients of the expansion, a_{ij}, are integers denoting approximately the number of each monomer types encountered in each PMMA–coronene configuration. Additional parameters, h_{ij}, define the height of the specific monomer relative to the one closest to the coronene plane. Explicitly these are:

s-PMMA, horizontal (experimental length):

$$U_{sh}(z) = a_{21}u_2(z+h_{21}) + a_{41}u_4(z+h_{41}) + a_{42}u_4(z+h_{42})$$

where a_{31} = 6, h_{31} = 0.0 Å, a_{41} = 4, h_{41} = 2.6 Å, a_{42} = 4, and h_{42} = 4.7 Å. When the theoretical s-PMMA helical pitch is used the corresponding potential is multiplied by a factor of 1.363.

s-PMMA, face-down:

$$U_{sf}(z) = a_{11}u_1(z+h_{11}) + a_{12}u_1(z+h_{12}) + a_{31}u_3(z+h_{31}) + a_{32}u_3(z+h_{32})$$

where a_{11} = 7, h_{11} = 0.0 Å, a_{12} = 1, h_{12} = 1.45 Å, a_{31} = 7, h_{31} = 2.3 Å, a_{32} = 1, and h_{32} = 5.2 Å.

i-PMMA, horizontal:

$$U_{ih}(z) = a_{11}u_1(z+h_{11}) + a_{12}u_1(z+h_{12}) + a_{21}u_2(z+h_{21}) + a_{31}u_3(z+h_{31})$$

where a_{11} = 6, h_{11} = 0.0 Å, a_{12} = 2, h_{12} = 1.3 Å, a_{31} = 2, h_{31} = 2.4 Å, a_{32} = 2, and h_{32} = 3.7 Å.

The fitting parameters for the modified Lennard–Jones composite potentials take the following values:

For U_{sh}: ε = 0.25209(2) kJmol^{-1}Å^{-2}, σ = 2.96414(2) Å, and C = 0.67282(5)

For U_{sf}: ε = 0.3014(1) kJmol^{-1}Å^{-2}, σ = 2.7495(1) Å, and C = 0.7093(3)

For U_{ih}: ε = 0.3401(1) kJmol^{-1}Å^{-2}, σ = 2.75851(9) Å, and C = 0.7236(2)

These primitive and composite potentials can be used to create mesoscopic models for the interaction of graphene and PMMA with regions of arbitrary tacticity.

5. Effective Stiffness of Polymer Matrix

A second spring is included in the analysis through which the elasticity of the polymer matrix is accounted for. This spring is in series with the corresponding VdW (first) spring. The equivalent stiffness of the two springs is given by

$$\frac{1}{k_{\mathrm{W}}}=\frac{1}{k_{\mathrm{VdW}}}+\frac{1}{k_{\mathrm{PMMA}}}$$

where the constants k_W, k_{VdW}, and k_{PMMA} are defined per unit area.

From the graph given in figure S6, to obtain the effective (second) spring length, L_0, we use the specific Young's modulus, E_{PMMA} and the Van der Waals spring constant, k_{VdW}. In addition, the experimental Winkler modulus, k_W, as obtained via the mathematical model (see section SI-1), is also used.

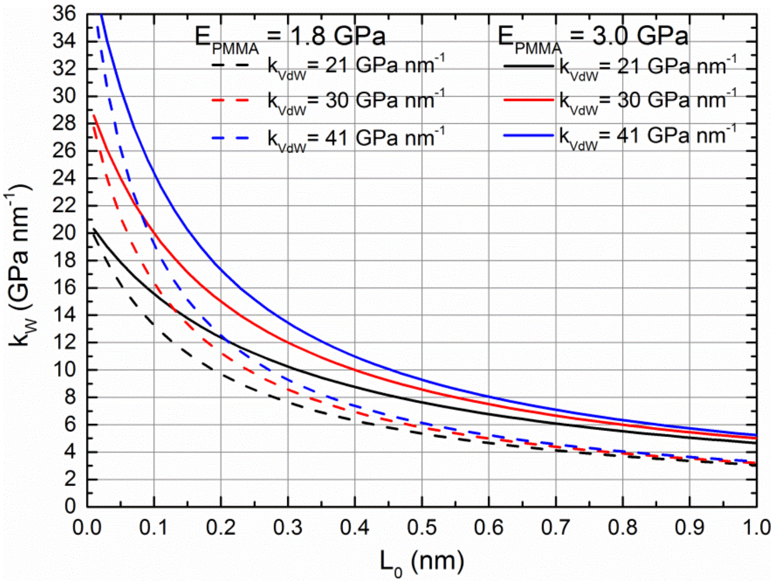

Figure S6. Family of curves corresponding to the range of Young's moduli for PMMA and the range of calculated VdW spring stiffnesses.

References

[1] R. J. Knops, E.W. Wilkes, Theory of Elastic Stability, Handbuch der Physik. pp. 125-301. Vol. VIa/3, Springer, Berlin (1973).

[2] S. P. Timoshenko, J.M. Gere, Theory of elastic stability, McGraw-Hill (1961).

[3] R. Huang, Kinetic wrinkling of an elastic film on a viscoelastic substrate, J. Mech. phys. Sol. 53 (2005) 63-89.

[4] Z. Y. Huang, W. Hong, Z. Suo, Nonlinear analyses of wrinkles in a film bonded to a compliant substrate. J. Mech. Phys. Sol. 53 (2005) 2101-2118.

[5] H. Jiang, D.-Y Khang, J. Song, Y. Sun, Y. Juang. J.A. Rogers, Finite deformation mechanics in buckled thin films on compliant supports. PNAS 104 (2007) 15607-15612.

[6] E. Puntel, L. Deseri, E. Fried, Wrinkling of a stretched thin sheet. J. Elasticity 105 (2011) 137-170.

[7] E. Cerda, L. Mahadevan, Geometry and physics of wrinkling. Phys. Rev. Lett. 90 (2003) 074302.3.

[8]L. Gong, I.A. Kinloch, R.J. Young, I. Riaz, R. Jalil, K.S. Novoselov, Advanced Materials 22 (2010) 2694-2697.

[9] T. M. G. Mohiuddin, A. Lombardo, R. R. Nair, A. Bonetti, G. Savini, R. Jalil, N. Bonini, D. M. Basko, C. Galiotis, N. Marzari, K. S. Novoselov, A. K. Geim, and A. C. Ferrari, Uniaxial strain in graphene by Raman spectroscopy: G peak splitting, Grüneisen parameters, and sample orientation, Phys. Rev. B 79 (2009) 205433.

[10] Tsoukleri, G.; Parthenios, J.; Papagelis, K.; Jalil, R.; Ferrari, A. C.; Geim, A. K.; Novoselov, K. S.; Galiotis, C., Subjecting a Graphene Monolayer to Tension and Compression. *Small* 2009, *5* (21), 2397-2402.

[11] Mohr, M.; Papagelis, K.; Maultzsch, J.; Thomsen, C., Two-Dimensional Electronic and Vibrational Band Structure of Uniaxially Strained Graphene from *ab initio* Calculations, *Phys. Rev. B* 2009, 80, 205410.
